# Supplementary material for: Meningeal lymphatics clear erythrocytes that arise from subarachnoid hemorrhage
Source: Nat Commun. 2020 Jun 22;11:3159. doi: 10.1038/s41467-020-16851-z (PMC7308412; doi:10.1038/s41467-020-16851-z)
Supplement: Supplementary file 1 — Supplementary Information [file 41467_2020_16851_MOESM1_ESM.pdf]

Supplementary Figures

Meningeal lymphatics clear erythrocytes that arise from subarachnoid hemorrhage

Chen et al.

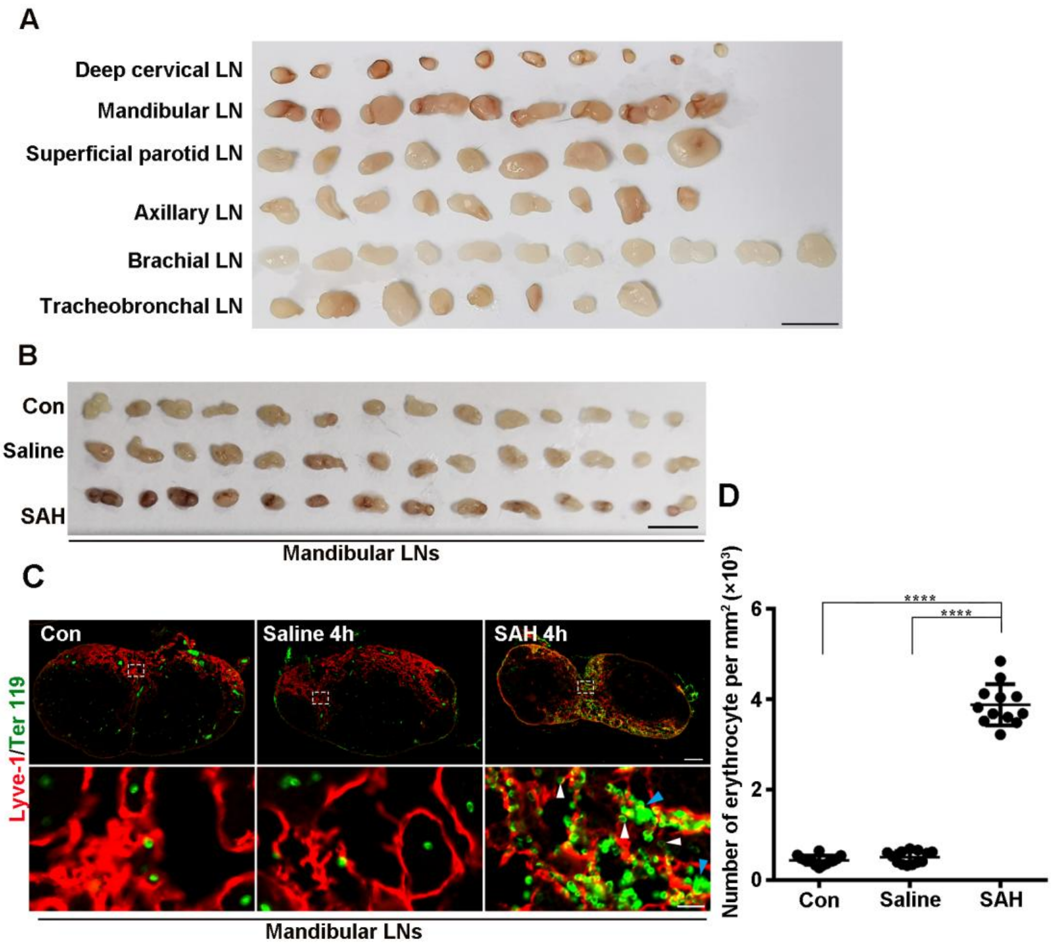

Supplementary Figure 1 Autologous blood injected into cisterna magna was specifically drained into deep cervical lymph node and mandibular lymph node. (A) Main lymph nodes in neck, forelimb and chest were isolated, the extravasated blood was drained to only dCLNs and mandibular LNs, the superficial parotid LNs also located in the superficial anterior neck, seems did not drain the extravasated blood. (B) The mandibular LNs of different groups at 4 hours (h) post injection of saline or autologous blood. (C) Representative images of erythrocytes in Lyve-1+ lymphatic sinus of mandibular LNs. n=2 independent experiments. Regions of interest in the top images are shown below. Scale bar, 200μm (top images) and 20μm (bottom images). White arrows, morphologically intact erythrocytes. Blue arrows, clusters of degraded membrane. (D) Quantification of the number of ter 119 labeled erythrocytes per mm^2 Lyve-1+ lymphatic sinus of mandibular LNs at 4 h post SAH induction (Con; n=14, Saline; n=13, SAH; n=12 mice, pooled from 2 independent experiments. *P*(Con

vs SAH) < 0.0001, *P*(Saline vs SAH) < 0.0001). All data are presented as mean values ± SD; one-way ANOVA with Turkey's multiple-comparison test, ****P < 0.0001. NS, not significant. Source data are provided as a Source Data file

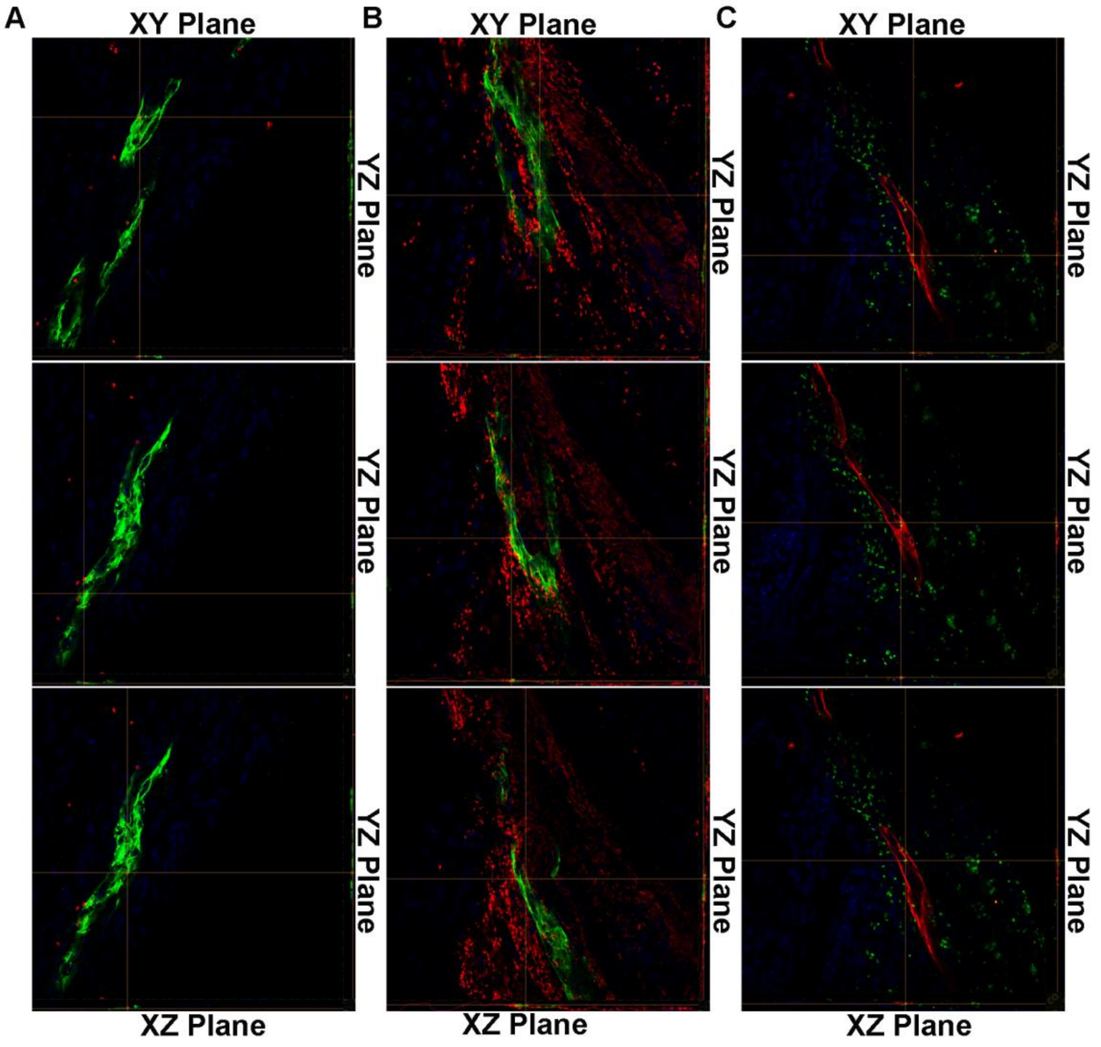

Supplementary Figure 2 Orthogonal view of Ter 119-positive erythrocytes and CFSE-labeled (ex vivo) erythrocytes within lymphatic vessels.

(A-B) Representative orthogonal views of meningeal lymphatics from a saline group (A) or SAH group (B) mice as shown in Fig 1E. (C) Representative orthogonal views of meninges from CFSE-labeled (ex vivo) erythrocytes injection group as shown in Fig. 2A. The orthogonal views show the Ter 119+ cells or CFSE-labeled erythrocytes of interest in the XY, XZ, and YZ planes co-localizing with Lyve-1+ lymphatic endothelial cells, suggesting these cells are indeed erythrocytes drained by meningeal lymphatic vessels.

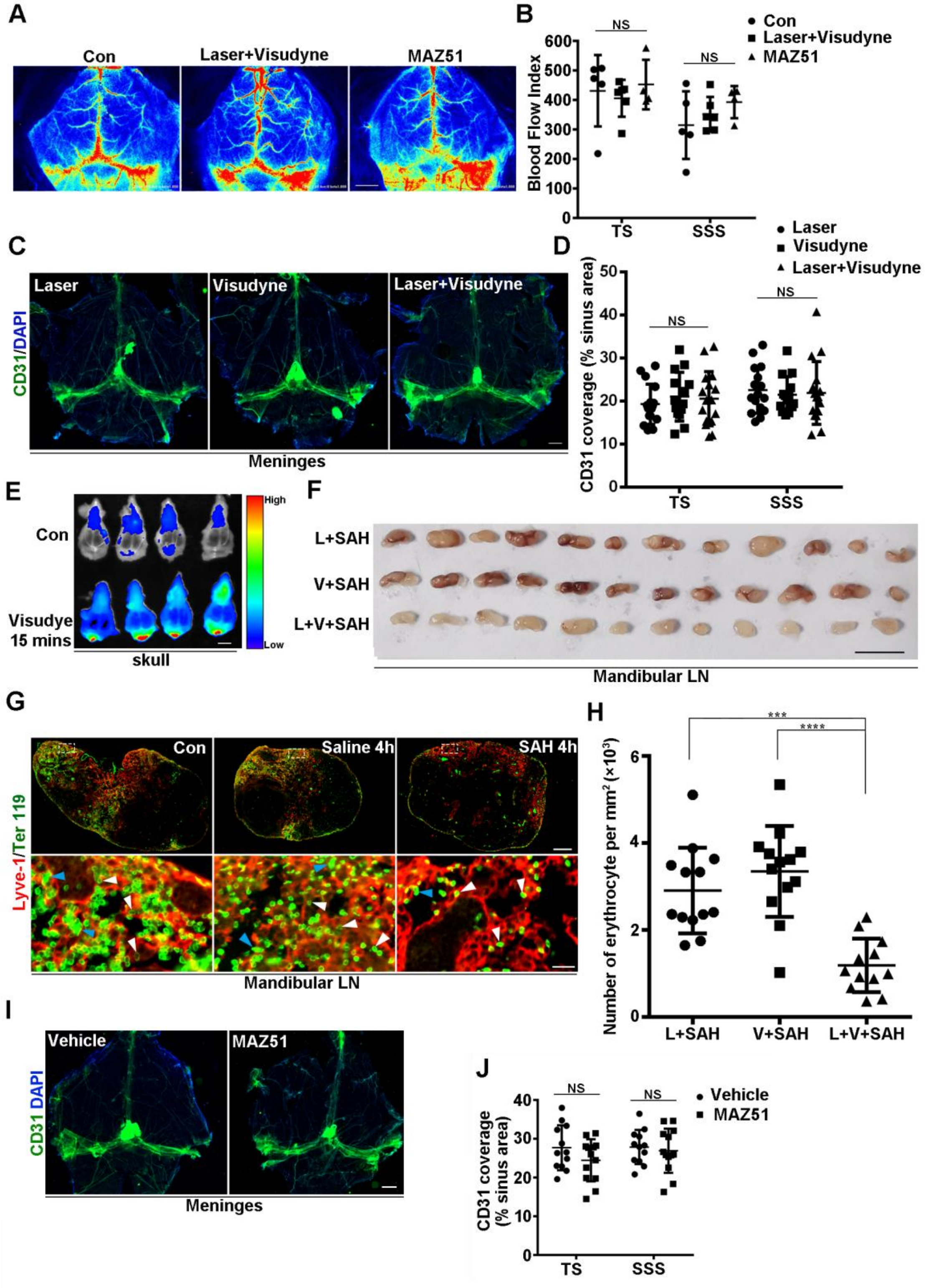

Supplementary Figure 3 The ablation of meningeal lymphatics and VEGFR3 blockage did not alter the cerebral blood flow and vasculature coverage on sinus. (A) Representative images of cerebral blood flow detected by laser speckle. Control group mice were not received any treatment before detection, Visudyne+Laser and MAZ51

group mice were detected at 7 days post lymphatics ablation and at 30 days post MAZ51 administration, respectively. Scale bar, 2 mm. (B) Quantification of the cerebral blood flow index on transverse sinus（TS） and superior sagittal sinus (SSS) (Con; n=5, Laser+Visudyne; n=6, MAZ51; n=4). (C) Representative images of CD31 labeled vasculature (green) on meninges of Laser only, Visudyne only and Visudyne+Laser group. Scale bar, 1 mm. (D) Quantification of CD31 positive vasculature coverage on TS and SSS. Laser/laser+Visudyne; n=16, Visudyne; n=14 mice, pooled from 2 independent experiments). (E) Fluorescence imaging of visudyne distribution on the skull 15 minutes after injection Scale bar, 5 mm. (F) Mandibular LNs of laser only, visudyne only and laser + visudyne group after 4 hours of SAH induction. The extravasated blood infused into mandibular LNs of laser + visudyne group were significantly lower the other groups. (G) Representative images of mandibular LNs labeling lymhatics (red) and erythrocytes (green). Regions of interest in the top images are shown below. Scale bar, 200μm (top images) and 20μm (bottom images). White arrows, morphologically intact erythrocytes. Blue arrows, degraded membrane. n=2 independent experiments. (H) Quantification of the number of erythrocytes in the Lyve-1+ lymphatic sinus of mandibular LNs. L+SAH/V+SAH; n=13, L+V+SAH; n=12, pooled from 2 independent experiments. P(L+SAH vs L+V+SAH)=0.0001, P(V+SAH vs L+V+SAH) < 0.0001. (I) Representative images of meningeal blood vasculature (CD31) of mice treated with vehicle or MAZ51. Scale bar, 1 mm. (J) Quantification of the CD31 coverage on the TS and SSS of mice treated with vehicle or MAZ51. Vehicle; n=12, MAZ51; n=13 mice. All data are presented as mean values ± SD; two-tailed unpaired Student’s t-test (J) or one-way ANOVA with Turkey’s multiple-comparison test (B, D, H), ***P < 0.001, ****P ＜ 0.0001. NS, not significant. Source data are provided as a Source Data file.
