# Supplementary material for: Meningeal lymphatics clear erythrocytes that arise from subarachnoid hemorrhage
Source: Nat Commun. 2020 Jun 22;11:3159. doi: 10.1038/s41467-020-16851-z (PMC7308412; doi:10.1038/s41467-020-16851-z)
Supplement: Supplementary file 2 — Reporting Summary [file 41467_2020_16851_MOESM2_ESM.pdf]

nature research

Corresponding author(s): Qianqian Liang & Yongjun Wang

Last updated by author(s): Mar 9, 2020

Reporting Summary

Nature Research wishes to improve the reproducibility of the work that we publish. This form provides structure for consistency and transparency in reporting. For further information on Nature Research policies, see Authors & Referees and the Editorial Policy Checklist .

Statistics

For all statistical analyses, confirm that the following items are present in the figure legend, table legend, main text, or Methods section.

n/a	Confirmed	
☐	☒	The exact sample size (*n*) for each experimental group/condition, given as a discrete number and unit of measurement
☐	☒	A statement on whether measurements were taken from distinct samples or whether the same sample was measured repeatedly
☐	☒	The statistical test(s) used AND whether they are one- or two-sided<br>*Only common tests should be described solely by name; describe more complex techniques in the Methods section.*
☒	☐	A description of all covariates tested
☐	☒	A description of any assumptions or corrections, such as tests of normality and adjustment for multiple comparisons
☐	☒	A full description of the statistical parameters including central tendency (e.g. means) or other basic estimates (e.g. regression coefficient) AND variation (e.g. standard deviation) or associated estimates of uncertainty (e.g. confidence intervals)
☐	☒	For null hypothesis testing, the test statistic (e.g. *F*, *t*, *r*) with confidence intervals, effect sizes, degrees of freedom and *P* value noted<br>*Give P values as exact values whenever suitable.*
☒	☐	For Bayesian analysis, information on the choice of priors and Markov chain Monte Carlo settings
☒	☐	For hierarchical and complex designs, identification of the appropriate level for tests and full reporting of outcomes
☒	☐	Estimates of effect sizes (e.g. Cohen's *d*, Pearson's *r*), indicating how they were calculated

Our web collection on statistics for biologists contains articles on many of the points above.

Software and code

Policy information about availability of computer code

Data collection	Image J bundled with 64-bit Java 1.8.0_112, Microsoft Excel 2007, BD Fortessa X20, Flow jo V10 software, EthoVision XT 12 (Noldus)
Data analysis	Graphpad Prism 6.0

For manuscripts utilizing custom algorithms or software that are central to the research but not yet described in published literature, software must be made available to editors/reviewers. We strongly encourage code deposition in a community repository (e.g. GitHub). See the Nature Research guidelines for submitting code & software for further information.

Data

Policy information about availability of data

All manuscripts must include a data availability statement. This statement should provide the following information, where applicable:

- Accession codes, unique identifiers, or web links for publicly available datasets
- A list of figures that have associated raw data
- A description of any restrictions on data availability

The raw data underlying Figs 1c, e, 2b, e, 3b-c, f, 4c-h, 5c, f-k, 6b-e, g-h, j, 7b-e and Supplementary Figs. 1d, 3b, d, h, j are available via a source data file submitted with this manuscript. All other data are available from the corresponding authors upon reasonable requests.

Field-specific reporting

Please select the one below that is the best fit for your research. If you are not sure, read the appropriate sections before making your selection.

Life sciences study design

All studies must disclose on these points even when the disclosure is negative.

Sample size	No sample-size calculations were performed. Sample size was determined according to previously publications and reviewers' recommendations.
Data exclusions	On principle, data were only excluded for failed experiments, reasons for which animals did not survive to the end of experiment.
Replication	For the all in vivo experiments, each experiment was repeated at least 2 times. The replication details are in the corresponding figure legends.
Randomization	No particular procedure was applied for randomization/ allocating C57BL/6J mice to the respective experimental groups.
Blinding	The investigator responsible for image analysis and data collection are blinded to group allocation

Reporting for specific materials, systems and methods

We require information from authors about some types of materials, experimental systems and methods used in many studies. Here, indicate whether each material, system or method listed is relevant to your study. If you are not sure if a list item applies to your research, read the appropriate section before selecting a response.

Materials & experimental systems

n/a	Involved in the study	
☐	☒	Antibodies
☒	☐	Eukaryotic cell lines
☒	☐	Palaeontology
☐	☒	Animals and other organisms
☒	☐	Human research participants
☒	☐	Clinical data

Methods

n/a	Involved in the study	
☒	☐	ChIP-seq
☐	☒	Flow cytometry
☒	☐	MRI-based neuroimaging

Antibodies

Antibodies used

Sources and usage details of all the antibodies used in the study are described in the methods section.
rabbit anti-Lyve-1 (1:1000; Abcam, Cat. No. ab14917), rat anti-Ly76 [Ter119] (1:500; Abcam, Cat. No. ab91113), rat anti-Ter 119 PE-conjugated (1:100; Cat.No. 12-5921-81, ebioscience), rat anti-Lyve-1 660 conjugated (1:200; 50-0443-80, ebioscience), hamster anti-Podoplanin (1:200; Abcam, Cat.No. ab11936), rabbit anti-Prox1 (1:100; AngioBio, Cat.No. 11-002P), rat ant-CD31 (1:100, abcam, Cat.No. ab7388). DylightTM 488 labeled goat anti-rabbit (1:200; KPL, Cat.No.072-03-15-06), DylightTM 488 labeled goat anti-rat (1:200; Cat.No.072-03-16-06, KPL), Alexa Fluor 546 goat anti-hamster (1:200, Invitrogen, Cat.No. A-21111), Alexa Fluor 488 goat anti-rat (1:1000, cell signaling technology, Cat.No. 4416S), Alexa Fluor 555 goat anti-rat (1:1000, cell signaling technology, Cat.No. 4417S), Alexa Fluor 555 goat anti-rabbit (1:1000, cell signaling technology, Cat.No. 4413S), rat anti-CD11b FITC-conjugated (1:100, 11-0112-82, ebioscience), rat anti-CD45 Percp Cy5.5-conjugated (1:100, 45-0451-82, ebioscience), rat anti-CD16/32 APC-conjugated (1:100, 558636, BD Bioscience) , rat anti-CD206 PE-conjugated (1:100, 12-2061-80, ebioscience), Rat IgG2b κ Isotype control FITC-conjugated (1:100, 11-4031-82, ebioscience) , Rat IgG2a κ Isotype control PerCP-Cy 5.5-conjugated (1:100, 45-4321-80, ebiosience), Rat IgG2b κ Isotype control PE-conjugated (1:100, 12-4031-82, ebioscience), Rat IgG2b κ Isotype control APC- conjugated (1:100, 553991, BD Bioscience).

Validation

rabbit anti-Lyve-1 https://www.abc am.cn/lyve1-antibody-ab14917.html
rat anti-Ly76 https://www.abcam.cn/ly76-antibody-ter-119-ab91113.html
rat anti-Ter 119 PE-conjugated https://www.thermofisher.com/cn/zh/antibody/product/TER-119-Antibody-clone-TER-119-Monoclonal/12-5921-81
rat anti-Lyve-1 660 conjugated https://www.thermofisher.com/cn/zh/antibody/product/LYVE1-Antibody-clone-ALY7-Monoclonal/50-0443-80
hamster anti Podoplanin https://www.abcam.cn/podoplanin--gp36-antibody-rtd4e10-ab11936.html
rabbit anti-Prox1 https://www.insightbio.com/productinfo/11-002P/AngioBio
rat anti-CD31 https://www.abcam.cn/cd31-antibody-mec-746-ab7388.html
DylightTM 488 labeled goat anti-rabbit https://us.vwr.com/store/supplier/id/0000018044/KPL?refineWord=072-03-15-06
DylightTM 488 labeled goat anti-rat https://us.vwr.com/store/supplier/id/0000018044/KPL?refineWord=072-03-16-06
Alexa Fluor 546 goat anti-hamster https://www.thermofisher.com/cn/zh/antibody/product/Goat-anti-Hamster-IgG-H-L-Cross-Adsorbed-Secondary-Antibody-Polyclonal/A-21111
Alexa Fluor 488 goat anti-rat https://www.cellsignal.com/products/secondary-antibodies/anti-rat-igg-h-l-alexa-fluor-488-conjugate/4416;jsessionid=nxK26ThvlpbG8rHDp__qviE1bKOhrrSbVLaUtKit?site-search-type=Products&N=4294956287&Ntt=alexa+fluor+488+goat+anti-rat+&fromPage=plp&_requestid=61191
Alexa Fluor 555 goat anti-rat https://www.cellsignal.com/products/secondary-antibodies/anti-rat-igg-h-l-alexa-fluor-555-conjugate/4417;jsessionid=1QH_ilseE-aVu-Zc56AJFhYy4CT4dVU27WUxjRsq?site-search-

type=Products&N=4294956287&Ntt=alexa+fluor+555+goat+anti-rat+&fromPage=plp&_requestid=60533
Alexa Fluor 555 goat anti-rabbit https://www.cellsignal.com/products/secondary-antibodies/anti-rabbit-igg-h-l-f-ab-2-fragment-alexa-fluor-555-conjugate/4413;jsessionid=gjul-ryLnG-3MkGVdqHFIzgK6_4dEEjEEqipJJSJ?site-search-type=Products&N=4294956287&Ntt=alexa+fluor+555+goat+anti-rabbit+&fromPage=plp&_requestid=60616
rat anti-CD11b FITC-conjugated https://www.thermofisher.com/cn/zh/antibody/product/CD11b-Antibody-clone-M1-70-Monoclonal/11-0112-82
rat anti-CD45 Percp Cy5.5-conjugated https://www.thermofisher.com/cn/zh/antibody/product/CD45-Antibody-clone-30-F11-Monoclonal/45-0451-82
rat anti-CD16/32 APC-conjugated https://www.bdbiosciences.com/cn/applications/research/b-cell-research/surface-markers/mouse/apc-rat-anti-mouse-cd16cd32-fc-iiiii-receptor-24g2/p/558636
rat anti-CD206 PE-conjugated https://www.thermofisher.com/cn/zh/antibody/product/CD206-MMR-Antibody-clone-MR6F3-Monoclonal/12-2061-80
Rat IgG2b κ Isotype control FITC-conjugated https://www.thermofisher.com/cn/zh/antibody/product/Rat-IgG2b-kappa-clone-eB149-10H5-Isotype-Control/11-4031-82
Rat IgG2a κ Isotype control PerCP-Cy 5.5-conjugated https://www.thermofisher.com/cn/zh/antibody/product/Rat-IgG2a-kappa-clone-eBR2a-Isotype-Control/45-4321-80
Rat IgG2b κ Isotype control PE-conjugated https://www.thermofisher.com/cn/zh/antibody/product/Rat-IgG2b-kappa-clone-eB149-10H5-Isotype-Control/12-4031-82
Rat IgG2b κ Isotype control APC- conjugated https://www.bdbiosciences.com/cn/reagents/research/antibodies-buffers/immunology-reagents/anti-mouse-antibodies/cell-surface-antigens/apc-rat-igg2b-isotype-control-a95-1/p/553991

Animals and other organisms

Policy information about studies involving animals; ARRIVE guidelines recommended for reporting animal research

Laboratory animals	Specific pathogen-free, C57BL/6 male mice (6~8-week-old) were purchased from Shanghai Model Organisms Center. Mice were housed in the animal facility with controlled habituation and temperature, on 12 h light:dark cycles, and fed with regular rodent's chow and sterilized tap water ad libitum. Mice were allowed to accommodate for two weeks before experimental procedures.
Wild animals	the study did not involve wild animals
Field-collected samples	the study did not involve samples from the field
Ethics oversight	All animal procedures were approved by Longhua Hospital - Animal Ethics Committee and were performed according to the Guiding Principles for the Care and Use of Laboratory Animals Approved by Animal Regulations of National Science and Technology Committee of China.

Note that full information on the approval of the study protocol must also be provided in the manuscript.

Flow Cytometry

Plots

Confirm that:

- [x] The axis labels state the marker and fluorochrome used (e.g. CD4-FITC).
- [x] The axis scales are clearly visible. Include numbers along axes only for bottom left plot of group (a 'group' is an analysis of identical markers).
- [x] All plots are contour plots with outliers or pseudocolor plots.
- [x] A numerical value for number of cells or percentage (with statistics) is provided.

Methodology

Sample preparation	Sample preparation listed in Methods.<br>Mice brains were dissected after transcardial perfusion by cold PBS, then minced into small pieces. Brain tissue was digested by collagenase A (1mg/ml, Sigma Aldrich, Cat.No. 10103578001) for 30 minutes at 37ḉ , then filtered by 70-μm nylon mesh cell strainers (BD bioscience). A cell suspension was made with 30% stock isotonic percoll (SIP) (GE, 17089109) and layered on the top of 70% SIP and then centrifuged at 500g at 25ḉ for 30 minutes without braking. Cells were collected from the 70%~30% SIP interphase and stained for live cells by Fixable Viability Dye eFluorTM 780 (Cat.No. 65-0865-18, ebioscience), extracellular markers with the following antibodies at a 1:100 dilution: rat anti-CD11b FITC-conjugated (11-0112-82, ebioscience), rat anti-CD45 Percp Cy5.5-conjugated (45-0451-82, ebioscience), rat anti-CD16/32 APC-conjugated (558636, BD Bioscience) and intracellular markers rat anti-CD206 PE-conjugated (12-2061-80, ebioscience). The corresponding isotype control antibodies that were used are as follow: Rat IgG2b κ Isotype control FITC-conjugated (11-4031-82, ebioscience) Rat IgG2a κ Isotype control PerCP-Cy 5.5-conjugated (45-4321-80, ebiosience), Rat IgG2b κ Isotype control PE-conjugated (12-4031-82, ebioscience), Rat IgG2b κ Isotype control APC- conjugated (553991, BD Bioscience).
Instrument	BD Fortessa X20

Software	Flow jo V10 software
Cell population abundance	No tests were performed to asses the purity of each population after sorting.
Gating strategy	Using the FSC/SSC gating, debris was removed by gating on the main cell population. Positivity threshold for each sample was defined on the basis of non-specific IgG treated negative control. Identical positivity threshold was applied to all samples.

☐ Tick this box to confirm that a figure exemplifying the gating strategy is provided in the Supplementary Information.
